# A framework to benchmark the quality of clinical assessment in a South African undergraduate medical programme

**DOI:** 10.4102/safp.v62i1.5030

**Published:** 2020-02-04

**Authors:** Hanneke Brits, Johan Bezuidenhout, Lynette J. van der Merwe

**Affiliations:** 1 Department of Family Medicine, Faculty of Health Sciences, Free State University, Bloemfontein, South Africa; 2 Department of Health Professions Education, Faculty of Health Sciences, Free State University, Bloemfontein, South Africa; 3 Department of Undergraduate Programme Management, Faculty of Health Sciences, University of the Free State, Bloemfontein, South Africa

**Keywords:** accreditation, assessment policies, assessment guidelines, clinical assessment, quality assurance in assessment, principles of quality assessment, undergraduate assessment

## Abstract

**Background:**

The outcome of the undergraduate medical programme is to produce clinically competent health care providers relevant for the South African context. Educational institutions find it hard to ensure the quality of assessments where competency must be assessed. This study aimed to compile an assessment framework that can be used to benchmark current assessment practices in the clinical phase of the undergraduate medical programme where competency must be certified.

**Methods:**

In this observational, descriptive study, qualitative data were gathered using the steps described by the World Health Organization for rapid reviews. Literature was searched, screened and selected before data were analysed and a framework was constructed.

**Results:**

Twenty-five official documents were included in the study. The framework addressed the three components of quality assessment, namely, accreditation, assessment and quality assurance. Assessors should attend to the principles of assessment, namely, validity, reliability, fairness, feasibility, educational effect and acceptability, but realise that no assessment meets all these criteria. The first step to ensure quality assessment is to identify a clear outcome. Assessment should be planned and aligned with this outcome.

**Conclusion:**

It is clear that clinical assessment is multidimensional and that no assessment is perfect. Programme accreditation, assessment practices and psychometrics can assist to improve the quality of assessment but cannot judge clinical competence. Using experienced assessors with a variety of assessment methods on a continuous basis is the proposed way to assess clinical competence. An assessment framework can assist to improve assessment, but it cannot guarantee quality assessment.

## Introduction

In South Africa, undergraduate medical training programmes are offered at nine accredited universities.^1^ The formal undergraduate medical training programme offered is the Bachelor of Medicine and Bachelor of Surgery (MBChB). Passing the final assessment of the MBChB programme enables a student to graduate and qualify as an entry-level medical practitioner.^2^ The main outcome of the MBChB programme is to produce clinically competent health care providers relevant for the South African context.^3^ This context is determined by the quadruple burden of disease (e.g. trauma, gastro-enteritis with dehydration, malnutrition, maternal and labour complications, human immunodeficiency virus and tuberculosis as well as lifestyle diseases),^4^ which, in turn, determines the required competencies to manage these conditions. Clinical assessment is unique because of integration of competencies and more than one possible correct approach to a problem.^5^ If clinical competence is assessed on the ‘does’ level of Miller’s pyramid,^6^ there will always be a compromise on reliability.^7^ Locally, the educational institution finds it hard to defend the quality of high-stakes competency assessments against validity, reliability and fairness. This study forms part of a larger study addressing quality of assessment in the clinical phase of the undergraduate medical programme.

Three components of quality in assessment have been described in the literature, namely, accreditation, assessment and audit.^8,9^ In this article, registration is added to accreditation, as the MBChB degree is a professional qualification that must be accredited and registered with various authorities. The term audit is replaced by quality assurance, as both these terms serve the purpose of improving quality; however, the term audit is usually used in the financial context, and quality assurance in education and other fields.^10^

To assess the quality of an assessment, it must be benchmarked against appropriate criteria. Benchmarking is described as the process of comparing standards with external criteria, with the aim of improvement.^11^ Clinical assessment should be benchmarked against best-practice evidence to ensure global relevance.^12^ Pangaro and Ten Cate recommended an assessment framework to benchmark assessment and competence against.^13^ In developing a framework, it is essential to clarify and/or define the terms or concepts that form the basis of the framework.^14^ McCall states that a good definition contains previously defined words, classifies and quantifies and has no counterexamples.^15^ The following terms and processes are used in this article.

### Framework

An assessment framework can be described as a ‘common language and mental model’ that guides assessors on what to look for in student assessment to maximise the reliability of the assessment. This framework also informs students and leadership on what to expect during assessment.^13^ It is important to realise that not all aspects of a framework necessarily apply to all assessments.^16^ A synthetic framework that integrates the domains of knowledge, skills and attitudes to ensure competence in real-life situations^13^ has been proposed for this study. Furthermore, for a framework to be of practical value, it should be simple enough to understand, remember and implement, while training and monitoring should form part of the implementation process.^13^

### Accreditation

Accreditation entails certification, which confirms that a programme and/or training facility is capable of fulfilling required specifications for a specific period. For instance, the South African Qualifications Authority (SAQA) accredits the providers who offer outcomes-based learning programmes that are aligned with registered unit standards and qualifications of the National Qualifications Framework (NQF).^17^

### Assessment

South African Qualifications Authority defines assessment as ‘a process used to identify, gather and interpret information and evidence against required competencies’ in order to make a judgement about a learner’s achievement.^18^ The University of the Free State (UFS) describes assessment as ‘the process of determining the value, significance, or extent of what students know, understand and can do with their knowledge as a result of their educational experience’.^19^ Assessment is therefore a comprehensive process, includes a variety of measurements for judging performance. The content and standard of assessment, types of assessment, assessment methods and principles of assessment are included under this concept.

### Quality assurance

Quality, standards and relevance are key elements of quality assurance.^9^ As far back as 1980, Donabedian defined quality in order to measure it.^20^ He concluded that quality is not one-dimensional but includes various aspects that should be agreed upon before the measurement. By applying the criteria of validity, reliability and defined concepts, quality has been described as ‘a situation when a set of inherent characteristics consistently fulfil the requirements of the organization’s … stakeholders’.^21^

### The context of undergraduate medical training and assessment

The duration of undergraduate medical training is between 5 and 6 years, and it is offered in three phases, namely, orientation, pre-clinical and clinical training.^1^ The MBChB programme at the UFS is a 5-year (10-semester) outcomes-based programme presented at the UFS and the accredited training platforms of the Free State Department of Health. The clinical phase of the MBChB programme comprises the second half of the third year, the fourth year and the fifth year (semesters 6–10) of undergraduate medical training. During this phase, students rotate through the different clinical disciplines and receive clinical exposure to patients, as well as theoretical training. Formative assessment takes place during rotations and summative assessment at the end of the academic year. To progress to the next year, a student must pass assessments in all disciplines, and both the clinical and theoretical components separately.^22^

A preliminary literature review was performed to identify frameworks, policies and criteria on quality assessment in the clinical phase of an undergraduate medical programme. At the institutional level, the assessment policy of the UFS sets minimum criteria for assessment of undergraduate students, which requires alignment with national policies and acts.^23^ The Health Professions Council of South Africa (HPCSA) also prescribes core competencies (adapted from CanMEDS) for undergraduate medical students.^24^ Internationally, the guidelines of the Association for Medical Education in Europe (AMEE) describe the importance of frameworks for clinical assessment,^13^ and the World Federation of Medical Education (WFME) has published basic standards for assessment in undergraduate training.^25^ Although these guides provide valuable information and principles, they are not directly transferable to the undergraduate South African and UFS medical training context where major emphasis is placed on clinical skills and clinical competence in the workplace. These guidelines focus on general programmes or assessment practices, and they do not apply specifically to assessment in the clinical phase of an undergraduate medical programme.

### Research question

What are the current regulations and policies as well as best evidence practices that inform quality assessment in the clinical phase of an MBChB programme?

### Objective

To compile a framework that can be used to benchmark current assessment practices based on official regulations and policies, and supported by best evidence practices to ensure defendable assessment in the clinical phase of the MBChB programme in South Africa.

In this article, a rapid review of the regulations and policy documents of the bodies that regulate the assessment and training of the MBChB programme at the UFS was used to formulate a framework for clinical assessment. This framework may be helpful to benchmark the quality of assessment in the current MBChB programmes in South Africa and beyond its borders.

## Methods

Qualitative data were gathered using a rapid review. No formal definition or uniform method is described to conduct a rapid review although a rapid review can be described as a simplified systematic review.^26,27^ Rapid reviews are typically used to inform decisions and compile guidelines.^28,29^ The single research question, narrow time frame, limited data sources, use of a single reviewer and minimal data synthesis^26^ justify the use of a rapid review in this article.

The World Health Organization (WHO) proposed seven steps to follow for a systematic review, which may be adjusted according to the specific needs for a rapid review.^29^ These steps are:

- needs assessment and topic selection
- protocol development with or without registration
- literature search
- screening and selection of the literature
- data extraction
- risk-of-bias and quality assessment of data
- knowledge synthesis.

In rapid reviews, some of these steps are commonly simplified or omitted, but the description of the method should not be compromised.^26^ The components most commonly adjusted are the use of one reviewer instead of two reviewers, not conducting quality assessments of included data and not using grey data.^27^

The following steps were used in this rapid review.

### Topic selection

The difficulty to defend the quality of clinical assessment in an undergraduate medical programme was identified as an area to investigate.

### Protocol development and registration

A protocol was developed before the study commenced. The protocol limited the inclusion of primary source documents to the following:

- official regulations and policy documents of the regulatory bodies responsible for assessment in undergraduate medical education at the UFS and South Africa
- international guidelines on clinical assessment issued by the WFME
- the AMEE guidelines on frameworks for clinical assessment.

The primary outcomes to investigate were:

- accreditation and registration
- assessment
- quality assurance.

### Systematic document search

The following search strategies were followed:

- No date limitations were placed on the documents included in the review.
- The search process was conducted from May to July 2019.
- An Internet data search was conducted.
- The *Health Professions Act, 56* of 1974^2^ was used as the original document source for national policies and the UFS assessment policy^23^ for institutional policies.
- These databases identified from the literature study were consulted: UFS official website, HPCSA, SAQA, Council on Higher Education (CHE), WFME and AMEE websites.
- The following words and/or phrases were searched: accreditation, assessment policies, assessment guidelines, clinical assessment, quality assurance in assessment, principles of quality assessment and undergraduate assessment. Searches were conducted with single words and phrases and the inclusion and exclusion of ‘AND’ and ‘OR’.
- Backward searching was performed using references and cross-references to related policies and regulations of the identified regulatory bodies.
- Forward searches of the literature entailed the search for related and updated information from the same documents or topics to ensure that all relevant information was identified.

### Screening and selection of articles

- Documents were screened for selection by a single review author. Documents initially found not to meet the outcome of the study were not included but saved separately. These documents were screened a second time to ensure that relevant data were not omitted. When in doubt, the study leader could assist with selection decisions.

### Data extraction

Documents were grouped according to the primary outcomes that were accreditation and registration, assessment and audit. The assessment category was subdivided into the following subcategories:

- assessment content and standards
- assessment types
- assessment methods
- principles of quality assessment.

A table displayed the specific outcomes that were addressed by each document included in the study.

### Limiting the risk-for-bias

This was omitted in this review, although care was taken to include all relevant documents by following the prescribed protocol. Document quality was not assessed as only official policies and regulations were included.

### Knowledge synthesis

For each category, the results of the review were summarised and discussed. This was supplemented by a secondary literature search to clarify concepts. The guidelines for framework development described by Pangaro and Ten Cate^13^ were then followed to display the results visually. Finally, recommendations were made for the implementation of the framework and research limitations were discussed.

### Quality and rigour of the data collection

To ensure the credibility of the data collected and to ensure that relevant documents were included in the document review, the protocol was strictly followed. National and international guidelines were added to enable the transferability of results to other institutions. The steps followed in the rapid review were described clearly to assess the dependability of the results.

### Ethical considerations

The study was registered and approved by the Health Sciences Research and Ethics Committee (HSREC) at the University of Free State (UFS-HSD 2019/0001/2304). As only documents in the public domain were used for this literature review and analysis, no permission was necessary.

## Results

The MBChB programme is offered under the legislation of the Department of Health and the Department of Education (previously the Ministry of Education). The *Health Professions Act, 56* of 1974^2^ was used as the original document source for national policies, the UFS assessment policy^23^ for local policies and the AMEE (https://amee.org/home) and WFME (https://wfme.org/) websites to benchmark against international assessment principles. Twenty-five documents were included in the rapid review. Table 1 displays the documents used in this rapid review.

**TABLE 1:** Primary documents used in document review.

Document	Accreditation or registration	Assessment				Quality assurance
		Assessment content and standard	Assessment types	Assessment methods	Principles of quality assessment	
South Arica. Council on Higher Education. *Higher Education Act 101* of 1997.^30^	√	-	-	-	-	-
South Africa. *Health Professions Act 56* of 1974 (Amended 2007). Education training and registration.^31^	√	-	-	-	-	-
South Africa. *Health Professions Act 56* of 1974 (Amended 2009). Regulations relating to the registration of students, undergraduate curricula and professional examinations in medicine.^2^	-	√	√	-	√	√
Health Professions Council of South Africa. Core competencies for undergraduate students in clinical associate, dentistry and medical teaching and learning programmes in South Africa 2014.^24^	-	√	-	-	-	-
Health Professions Council of South Africa. Accredited facilities. 2019.^1^	√	-	-	-	-	-
Health Professions Council of South Africa. Professional Boards. 2019.^32^	√	-	-	-	-	-
South African Qualifications Authority. *South African Qualifications Authority Act 58* of 1995.^33^	√	-	-	-	-	-
South African Qualifications Authority. The National Qualifications Framework Curriculum Development. 2000.^34^	-	-	√	-	√	-
South African Qualifications Authority. National Qualifications Framework and the Standards setting. 2003.^35^	-	-	-	-	√	√
South African Qualifications Authority. Criteria and Guidelines for Assessment of NQR Registered Unit standards and Qualifications. 2001.^36^	-	-	-	-	-	-
South African Qualifications Authority. Guidelines for integrated assessment. 2005.^37^	-	√	√	-	√	-
South African Qualifications Authority. National Policy and Criteria for Designing and Implementing Assessment for NQF Qualifications and Part-Qualifications and Professional Designations in South Africa. 2014.^18^	-	-	√	√	-	-
South Africa. National Qualifications Framework. *National Qualifications Framework Act 67* of 2008.^38^	√	-	-	-	-	-
University of the Free State. Teaching-Learning Policy. 2008.^39^	-	-	√	√	√	-
University of the Free State. Quality assurance policy. 2009.^40^	-	-	-	-	-	√
University of the Free State. Guidelines for the implementation of external moderation. 2009.^41^	-	-	-	-	-	√
University of the Free State. Assessment policy on the UFS coursework learning programme. 2016.^23^	-	-	√	√	√	√
University of the Free State. General rules for undergraduate qualifications, postgraduate diplomas, Bachelor Honours degrees, Master’s degrees, Doctoral degrees, Higher Doctorates, Honorary degrees and the Convocation. 2019.^19^	-	-	√	-	√	-
University of the Free State. Faculty of Health Sciences. Rule book. School of Clinical Medicine. Undergraduate Qualifications. 2019.^22^	-	√	√	-	√	-
University of the Free State. School of Clinical Medicine. Undergraduate programme management. 2019. SOP Quality assurance.^42^	-	-	-	-	-	√
World Federation for Medical Education. 2015. Standards. Basic Medical Education.^25^	-	-	√	√	√	√
World Federation for Medical Education. Accreditation. 2017.^43^	√	-	-	-	-	-
Pangaro L, Ten Cate O. AMEE Guide No. 78.^13^	-	√	√	√	√	-
Tavakol M, Dennick R. AMEE Guide 119.^44^	-	-	-	-	√	-

NQR, National Qualification Register; NQF, National Qualification Framework; UFS, University of the Free State; SOP, Standard Operating Procedures; AMEE, Association for Medical Education in Europe.

### Accreditation and registration

According to the *Higher Education Act*, the Ministry of Education must oversee and take responsibility for norms and standards in higher education.^30^ To assist with this task, the Minister of Education and Training established SAQA as a juristic person who must implement the objectives of the NQF.^18^ The NQF was established under the *SAQA Act* to classify, register, publish and articulate approved national qualifications.^33^ Medical training is addressed under the sub-framework for higher education. The CHE, as the Quality Committee for Higher Education as provided for in the *Higher Education Act*, oversees the quality of training and assessment in higher education. The quality committee must register appropriate professional bodies (in this case, the HPCSA) to ensure that qualifications meet the requirements for professional registration. The quality committees make recommendations to SAQA to register higher education qualifications.^38^

The *Health Professions Act* makes provision for appointing professional councils (in this case, the HPCSA) to establish professional boards. The Medical and Dental Board is responsible for overseeing undergraduate medical training, as well as registering health professionals under this act.^31^ The HPCSA is responsible for accrediting universities and health care training in South Africa.^38^

Because of globalisation and the increased demand for accountability in health care, the WHO and the WFME worked together on documents for the accreditation of health training institutions worldwide. The WFME gives ‘recognition status’ to an accrediting agency that meets international standards.^43,45^

### Assessment

Four components of assessment were identified, namely, assessment content and standards, assessment types, assessment methods and principles of assessment.

#### Assessment content and standards

An assessment to ensure a competent practitioner must include elements of knowledge, skills and values.^2,18,23,27^ South African Qualifications Authority describes knowledge as foundational competence, skills as practical competence and values as reflective competence. It also emphasises the importance of assessing prior learning, and that assessment must include content to identify and stimulate further learning.^34^ Assessment of values, also described as core competencies, soft skills or critical cross-field competencies, has been prescribed as components of assessment in different documents.^18,19,24,34^ Critical cross-field competencies identified were problem-solving, critical thinking, teamwork, responsibility, data management, effective communication and effective use of resources.^34^ The core competencies for a health care practitioner include being a professional, a communicator, collaborator, leader and manager, health advocate and scholar.^24^ Assessment standards are the minimum criteria that must be achieved to pass an assessment. These standards include criteria for content and difficulty, and should be reasonable, defensible and fair. Students and assessors must know all the required standards before the assessment.^35^ The MBChB programme is registered on NQF level 8.^35^ Although there is no perfect passing score, the UFS sets the pass mark at 50%.^23^ In Clinical Medicine, students must pass both theoretical and clinical assessments separately in order to progress.^2,22^ None of the documents addressed specific standard setting methods or processes.

#### Assessment types

Different types of assessment applicable to medical assessment were identified from the document review, namely, formative assessment, integrated assessment and summative assessment.^2,18,19,37,39^ Some of these types may overlap or be inclusive of each other; for example, integrated assessment may take place during formative and/or summative assessment.

Formative assessment is described as a series of assessments that occur during the learning and training process.^18,23^ The purpose of formative assessment is to support learning, identify learning needs and accumulate marks.^23^

Summative assessment is the assessment that takes place after learning. The aim of summative assessment is to award grades and to validate performance and competence.^18,23^ Integrated assessment is described as ‘assessment that permits the learner to demonstrate applied competence’ using different methods of assessment.^18,34^ Integrated assessment may occur at any time during the learning process.

#### Assessment methods

Theoretical, practical and integrated assessment methods were described, and they relate to the aim or outcome of the assessment. Theoretical assessments include multiple-choice questions, modified essay questions or short-answer questions, as well as long questions. Oral examinations can be used to test knowledge or to combine knowledge with communication skills. Clinical assessments include unobserved long cases, mini clinical evaluation exercises (mini-CEX), objective structured clinical examinations (OSCEs) and direct observation of clinical practice (DOPS). Integrated assessment methods include portfolios, logbooks, elective reports and workplace-based assessments, as well as feedback from stakeholders.^19,23,25^

#### Principles of quality assessment

From the UFS general rules^19^ and assessment policies,^23^ which are aligned with the *Higher Education Acts*, the *NQF Act* and, by implication, the *Health Act*, the following principles were identified:

- Assessment should be an integral part of curriculum planning and must be aligned with outcomes.
- Assessment should be performed on the appropriate NQF level in accordance with programme registration.
- All assessments should be planned to cover all assessment domains.
- Assessment takes place in a system and must be planned accordingly.
- In order to be a quality assessment, each of these assessments should fulfil criteria for validity, reliability, transparency, fairness and practicability.
- Moderation should form part of overall, as well as individual, assessments.
- There should be accountability for each assessment, with evidence that the assessment was moderated.

An assessment can be considered credible if the criteria for fairness, validity, reliability and practicability have been met.^36^

### Quality assurance

Quality assurance policies are essential for ensuring that specifications and standards are maintained.^23^ This article focusses on quality assurance applicable to assessment and addresses moderation, benchmarking and security of the assessment process. Moderation is guided by moderation policies.^41^ It is a process that involves a professional judgement of the validity, reliability and fairness of the assessment and involves students, assessors and external stakeholders. The WFME sets global standards for assessment that serve as benchmarks against which those responsible for medical education can evaluate their activities.^25^

## Discussion

All the primary documents necessary for the rapid review were available in the public domain on the identified websites. Information in these documents was aligned with each other. Many cross-references to other documents were found in source documents. By comparing the information in the respective documents, it was found that there was no contradiction in the documents. The data included in the rapid review can therefore be considered representative and appropriate for the purpose of this study.

The three components of quality assessment, namely, accreditation and registration, assessment and quality assurance, should from part of an assessment framework to benchmark current assessment. The inclusion of best-practice evidence in the framework will make the framework globally relevant.^12^

### Accreditation and registration

Accreditation and registration is usually not a problem for training facilities in South Africa as the HPCSA conducts regular site visits and requires annual progress reports from training facilities to ensure compliance with accreditation and registration requirements.^46^ For the MBChB programme, the following must be in place:

- Accreditation of the training provider and the qualification by the HPCSA.
- Training may take place only at a university registered with the Department of Education.
- The qualification must be registered with SAQA.
- All students in the MBChB programme must be registered with the HPCSA.

A recommendation of the 2010 Ottawa Conference was to develop criteria for accreditation of international medical educational programmes.^47^ In response, the WHO and WFME developed international accreditation criteria. The WFME awarded ‘recognition status’ to the HPCSA as the accrediting body in South Africa; all training programmes accredited by the HPCSA will therefore have internationally accredited status.^43^

### Assessment

Assessment in medical education is complex and includes various stakeholders, each with their own expectations. These stakeholders include students, teachers, lecturers, educational institutions, health care systems, regulatory bodies and patients.^47^ A competent health care practitioner who can integrate knowledge, skills and attitudes relevant to the South African context is the ultimate outcome of the outcomes-based medical curriculum. This competency must be observable and measurable to certify the student as competent. Competency is best assessed on the ‘Does’ level according to Miller’s pyramid.^6^ The overarching term of workplace-based assessment may be a solution to assess knowledge skills, behaviour, attitude and self-reflection in real-life situations.^48^ In spite of the advantages of workplace-based assessment, Miller states that, ‘no single assessment method can provide all the data required for judgement of anything as complex as the delivery of professional services by a successful physician’.^6^ This is echoed when researchers warned against the use of a single assessment when pass or fail decisions have serious implications, such as for registration or licencing.^49^ Assessment should be a continuous process with many data points that can be taken into consideration to make an informed judgement on competence.

The quality of clinical assessment can be improved if attention is given to the following assessment principles:

- Validity: Content validity can be improved with blueprinting of individual as well as overall assessments, and construct validity with appropriate assessment methods.
- Reliability: Reliability can be improved by training assessors, enhancing the quality of questions and mark sheets, and by increasing the number of stations or questions per assessment.
- Fairness: Although all assessment cannot be equal, there should be no discrimination against any student, assessor or patient. It is also important that assessment should be conducted according to the NQF level that the programme is registered for.
- Feasibility: All assessments should be realistic, practical and sensible in the context where they take place. This can be achieved by careful planning and consideration of all resources.
- Educational effect: Assessment should promote learning through study for assessments, or making use of workplace-based assessment and constructive feedback.
- Acceptability: All stakeholders, including patients, students and the educational institution, should be satisfied with the assessment. This can be achieved through transparency and keeping all stakeholders informed.

### Quality assurance

Moderation is a quality assurance process that confirms that the assessment is valid and reliable and meets minimum standards.^50^ Moderation should form part of the overall assessment in the MBChB programme, as well as of each assessment. Moderation can be conducted internally and/or externally, and should take place before and after assessments. An external moderator should moderate all high-stakes examinations.^40,41^ The aim of moderation is to check consistency and standards.^12^ Benchmarking is also part of the moderation process as the aim is quality improvement.

Limitations of the study: Although the rapid review was performed according to the protocol, the risk-for-bias and quality of documents were not evaluated by a second reviewer. These results may not be 100% transferable to all MBChB programmes as different universities have different assessment policies and methods.

The complexity of clinical assessment warrants that assessment be ‘evaluated on a programmatic level’ rather than on individual assessment level, as no individual assessment meets all the criteria of validity, reliability, educational impact, acceptability and cost.^7^

## Conclusion

This rapid review attempted to develop a framework to benchmark the quality of assessment in the clinical phase of an undergraduate medical programme. As a first step, all stakeholders should be aware of the outcome of the programme. All assessment and training in the MBChB programme must be aligned with the outcome of the programme, namely, to produce a competent medical practitioner who can integrate knowledge, skills and attitudes relevant to the South African context.

Open-mindedness is essential during the assessment process. Programme accreditation, assessment practices and psychometrics can assist to improve the quality of assessment but cannot judge clinical competence. Experienced assessors, using a variety of assessment methods on a continuous basis, is the proposed way to go. By implementing quality assurance processes, institutions can ensure that specifications and standards are maintained and improved, and that they are globally competitive. It is clear that clinical assessment is multidimensional and that no assessment is perfect. An assessment framework can assist to improve assessment, but it cannot guarantee quality assessment.

**FIGURE 1:**
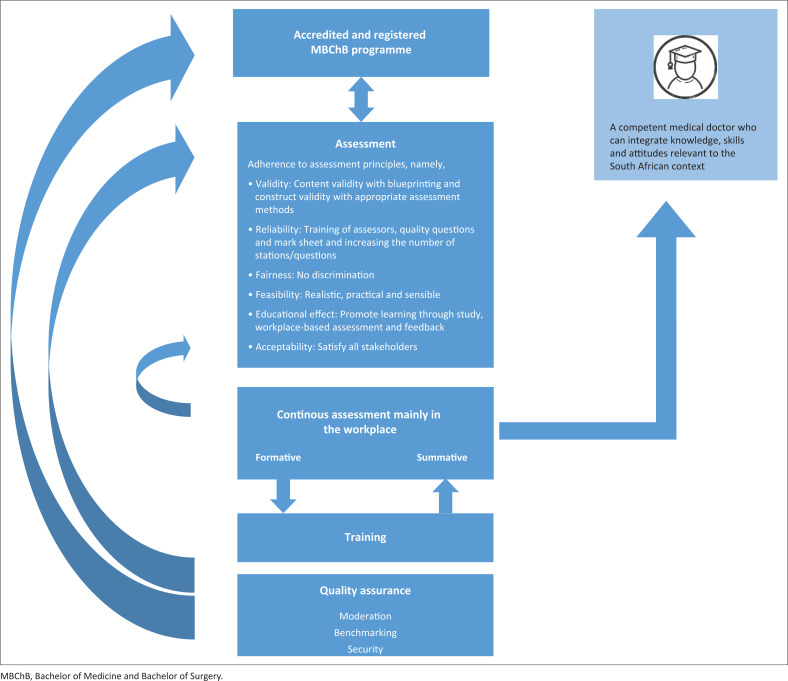
Schematic display of the framework to measure the quality of assessment in the clinical phase of an undergraduate medical programme.

Figure 1 is a schematic display of the framework for measuring the quality of assessment in the clinical phase of an undergraduate medical programme.
